# MXRA5 Is a Novel Immune-Related Biomarker That Predicts Poor Prognosis in Glioma

**DOI:** 10.1155/2021/6680883

**Published:** 2021-06-09

**Authors:** Jin-Zhang Sun, Jin-Hao Zhang, Jia-Bo Li, Feng Yuan, Lu-Qing Tong, Xu-Ya Wang, Lu-Lu Chen, Xiao-Guang Fan, Yi-Ming Zhang, Xiao Ren, Chen Zhang, Sheng-Ping Yu, Xue-Jun Yang

**Affiliations:** ^1^Department of Neurosurgery, Tianjin Medical University General Hospital, Tianjin 300052, China; ^2^Laboratory of Neuro-Oncology, Tianjin Neurological Institute, Tianjin 300052, China; ^3^Department of Neurosurgery, Affiliated Jinling Hospital, Medical School of Nanjing University, Nanjing 210002, China; ^4^Department of Neurosurgery, The First Affiliated Hospital, Zhejiang University School of Medicine, Hangzhou 310003, China

## Abstract

**Background:**

Glioma is the most common primary intracranial tumor and is associated with poor prognosis. Identifying effective biomarkers for glioma is particularly important. MXRA5, a secreted glycoprotein, is involved in cell adhesion and extracellular matrix remodeling and has been reported to be expressed in many cancers. However, the role and mechanism of action of MXRA5 in gliomas remain unclear. This study was aimed at investigating the role of MXRA5 at the transcriptome level and its clinical prognostic value.

**Methods:**

In this study, RNA microarray data of 301 glioma patients from the Chinese Glioma Genome Atlas (CGGA) were collected as a training cohort and RNA-seq data of 702 glioma samples from The Cancer Genome Atlas (TCGA) were used for validation. We analyzed the clinical and molecular characteristics as well as the prognostic value of MXRA5 in glioma. In addition, the expression level of MXRA was evaluated in 28 glioma tissue samples.

**Results:**

We found that MXRA5 expression was significantly upregulated in high-grade gliomas and IDH wild-type gliomas compared to controls. Receiver operating characteristic (ROC) analysis showed that MXRA5 is a potential marker of the mesenchymal subtype of glioblastoma multiforme (GBM). We found that MXRA5 expression is highly correlated with immune checkpoint molecule expression levels and tumor-associated macrophage infiltration. High MXRA5 expression could be used as an independent indicator of poor prognosis in glioma patients.

**Conclusion:**

Our study suggests that MXRA5 expression is associated with the clinicopathologic features and poor prognosis of gliomas. MXRA5 may play an important role in the immunosuppressive microenvironment of glioma. As a secreted glycoprotein, MXRA5 is a potential circulating biomarker for glioma, deserving further investigation.

## 1. Introduction

Gliomas account for the majority of primary malignant brain tumors in adults, and GBM is the most invasive and incurable type, which is characterized by a high recurrence rate and a high fatality rate [1, 2]. Despite the improvement of conventional treatments, including surgical resection and radiotherapy with concomitant temozolomide chemotherapy, the treatment of GBM remains a tremendous challenge [3, 4]. Recently, immunotherapies have changed cancer treatment strategies, especially the discovery of the intracranial lymphatic system, which has brought a new theoretical basis for brain tumor immunotherapy [5, 6].

Matrix-remodeling associated protein 5 (MXRA5), a secreted glycoprotein, is a member of the MXRA protein family that participates in cell adhesion and extracellular matrix (ECM) remodeling [7]. This protein contains 7 leucine-rich repeats and 12 immunoglobulin-like C2-type domains related to perlecan. MXRA5 is expressed in primates and some mammals but not in rats or mice [8]. MXRA5 mRNA is upregulated in human chronic ischemic myocardium and chronic kidney disease [9, 10]. In addition to ECM remodeling, MXRA5 has been found to be involved in tumorigenesis. For instance, somatic mutations of MXRA5 are observed in patients with non-small-cell lung cancer (NSCLC) [11]. MXRA5 protein is aberrantly expressed in colorectal cancer (CRC) tissues and serves as a biomarker for the early diagnosis of CRC [12].

However, little information is available concerning MXRA5 expression in glioma. Therefore, in the present study, we aimed to conduct a comprehensive analysis to explore the molecular and clinical characteristics of MXRA5 in glioma. We employed the CGGA mRNA microarray data as a training cohort, and our findings were validated in TCGA RNA-seq dataset successfully. We found that MXRA5 mRNA and protein expression was upregulated in glioma, especially GBM, and was an unfavorable prognostic biomarker for patients with glioma. This is the first integrative study molecularly and clinically characterizing MXRA5 expression in whole WHO-graded gliomas.

## 2. Materials and Methods

### 2.1. Patients and Tissue Samples

Gene expression microarray data of 301 glioma samples, ranging from WHO grade II to grade IV, were obtained from the CGGA (http://www.cgga.org.cn). The RNA-seq data of 702 glioma samples, ranging from WHO grade II to grade IV, were acquired from TCGA (http://cancergenome.nih.gov/). Twenty-eight glioma tissue samples were obtained from the Department of Neurosurgery, Tianjin Medical University General Hospital (1 grade I case, 7 grade II cases, 7 grade III cases, and 13 grade IV cases; grades were determined according to the 2016 WHO classification of tumors of the central nervous system). Written informed consent was obtained from the patients or their families. This study was conducted in compliance with the principles of the Helsinki Declaration and approved by the Ethics Committee of the General Hospital of Tianjin Medical University.

### 2.2. Cell Lines and Culture

A172 and TJ905 glioma cell lines were purchased from the Chinese Academy of Sciences Cell Bank (China). The A172 glioma cell line was cultured in Dulbecco's modified Eagle's medium (DMEM, Gibco, USA). The TJ905 glioma cell line was cultured in DMEM/F-12 (1 : 1) medium (Gibco, USA). Cells were not contaminated with mycoplasma. All cell culture media were supplemented with antibiotics (100 U/mL penicillin-streptomycin, Gibco, USA) and 10% fetal bovine serum (FBS, Gibco, USA) and incubated in 5% CO_2_ at 37°C.

### 2.3. Online Analysis of MXRA5 mRNA Expression in Tumors and Cell Lines

MXRA5 mRNA expression in tumors and normal tissues was explored by Gene Expression Profiling Interactive Analysis (GEPIA, http://gepia.cancer-pku.cn/ index.html). MXRA5 mRNA expression in cell lines was explored in the Cancer Cell Line Encyclopedia (CCLE, https://portals. http://broadinstitute.org/ccle).

### 2.4. Immunohistochemistry (IHC) and Immunofluorescence (IF) Staining

IHC and IF staining were performed as previously described by Li et al. [13]. Paraffin-embedded glioma tissues were used for IHC. A172 and TJ 905 glioma cell lines were used for IF staining. The cells were seeded in 24-well plates (1 × 10^4^ cells/well in 1 mL of medium) and incubated for 24 hours. The paraffin-embedded glioma sections were incubated with a MXRA5 primary antibody (ab99463, Abcam, USA, 1 : 100 dilution). Markers of IHC were detected with a two-step detection kit (PV-9003, ZSGB-Bio, Beijing, China). An Alexa Fluor^®^594-labeled anti-goat antibody (ZF-0517, ZSGB-Bio, 1 : 500) was added to the cell samples. The nuclei were stained with DAPI (Sigma-Aldrich). Images were obtained with a VANOX microscope (Olympus, Japan). IHC staining was assessed by the sum of the intensity and quantity scores. The intensity score was graded as 0 (no staining), 1 (light brown staining), 2 (brown staining), or 3 (dark brown staining). The quantity score was graded as 0 (no staining), 1 (1–25%), 2 (26–50%), 3 (51–75%), or 4 (>75%). The samples were defined as negative (0-2) or positive (>2) based on the final score. The stained sections were blindly evaluated by three independent pathologists who had no knowledge of the clinical information.

### 2.5. Quantitative Real-Time PCR (RT-qPCR) Analysis

The TRIzol^®^ reagent (Invitrogen, USA) was used to extract total RNA from glioma tissues and cell lines according to the manufacturer's instructions. The obtained RNA was reverse transcribed into cDNA using the First Strand cDNA Synthesis Kit (Promega, USA). RT-PCR was performed based on the Fast SYBRTM Green Master Mix according to the manufacturer's protocol (Promega, USA). The primer sequences designed for MXRA5 (NM_015419.4) were 5′-TCCAAGAGGATGATCGACGC-3′ (forward) and 5′-TGTGTTCAATCTTGGCCGGT-3′ (reverse). GAPDH was applied as an endogenous control. The primer sequences for GAPDH were 5′-TCACTGGCATGGCCTTCCGT-3′ (forward) and 5′-CTTACTCCTTGGAGGCCAT-3′ (reverse). Amplification was performed with 45 cycles of PCR in the MJ Real-Time PCR Detection System (Bio-Rad, USA). For relative quantification, 2^-*ΔΔ*CT^, which was calculated by subtracting the CT values of the control gene from the CT values of MXRA5, was used to determine the relative expression levels.

### 2.6. Statistical Analysis

The overall survival differences between groups were estimated by Kaplan-Meier analysis. Cox regression analysis was performed with SPSS statistical software (version 19). One-way ANOVA was used to test for differences among at least three groups. Student's *t*-test was used to determine differences in comparisons between two groups. The Pearson *χ*^2^ test was used to evaluate the correlation between MXRA5 expression level and clinicopathological parameters. Most figures and statistical analyses were performed with several packages (ggplot2, pROC, clusterProfiler, enrichplot, circlize, and corrgram) based on the R language version 3.6.2 (http://www.r-project.org) and GraphPad Prism 8.0. *p* < 0.05 was considered statistically significant.

## 3. Results

### 3.1. MXRA5 mRNA Expression in Glioma and Other Cancers

**Figure 1:**
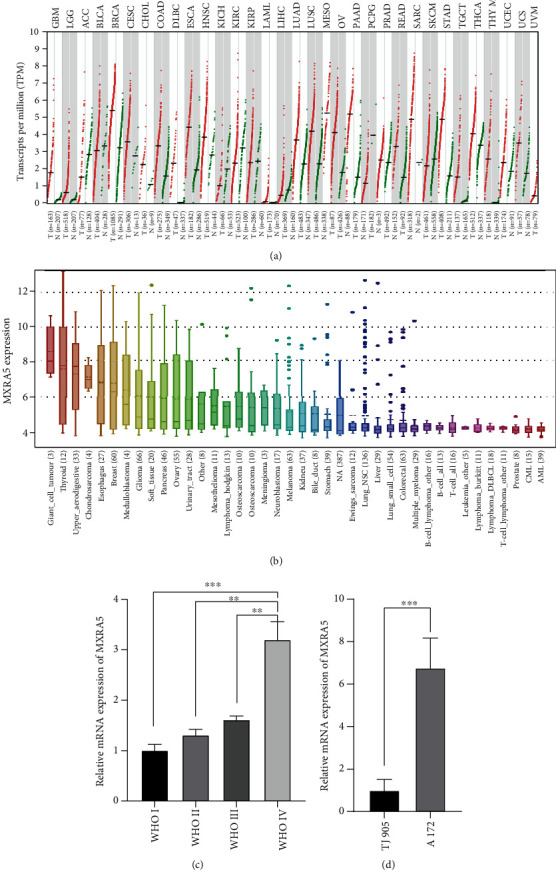
MXRA5 mRNA expression profile. (a) MXRA5 expression profiles across all tumor samples in TCGA dataset. (b) MXRA5 mRNA expression in 1019 cancer cell lines. (c, d) RT-qPCR analysis of MXRA5 in glioma tissue (c) and cell lines (d). ∗∗ and ∗∗∗ indicate *p* < 0.05 and *p* < 0.01, respectively.

In glioma and many malignancies, MXRA5 is more highly expressed in tumor tissues than in normal tissues (Figure 1(a)). To determine the expression of MXRA5 in cancer cells, we evaluated MXRA5 expression in the CCLE with 1019 cancer cell lines. We found that MXRA5 expression was higher in glioma than in most other cancers (Figure 1(b)). MXRA5 mRNA expression was upregulated in GBM compared to lower-grade gliomas (LGG), which was verified by RT-qPCR in twenty-eight glioma samples from Tianjin Medical University General Hospital (Figure 1(c)). To further verify the expression level of MXRA5 in glioma cell lines, we performed RT-qPCR with A172 cells and TJ905 cells (Figure 1(d)).

### 3.2. MXRA5 Is Highly Expressed in GBM and IDH Wild-Type Glioma

**Figure 2:**
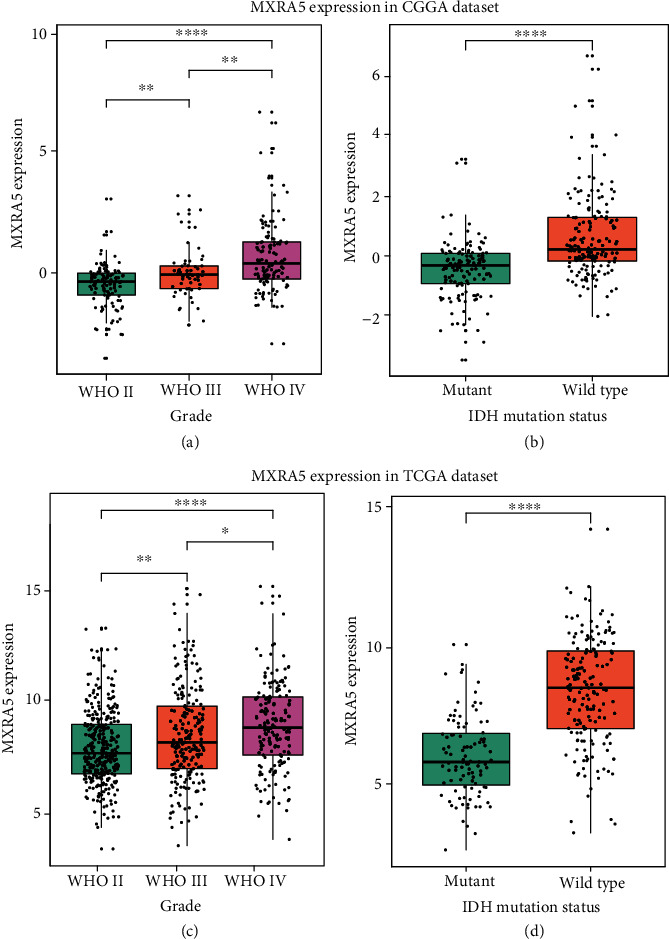
Comparison of MXRA5 mRNA expression levels in CGGA and TCGA cohorts with different WHO grades (a, c) and IDH mutation statuses (b, d). MXRA5 was significantly increased in WHO IV and IDH wild-type gliomas in the CGGA and TCGA datasets. ∗, ∗∗, and ∗∗∗∗ indicate *p* < 0.05, *p* < 0.01, and *p* < 0.0001, respectively.

MXRA5 mRNA expression was analyzed according to the WHO grading system in glioma. In the CGGA dataset, the expression of MXRA5 was significantly higher in GBM than in LGG (Figure 2(a)). This result was well validated in TCGA dataset (Figure 2(c)), which indicated that MXRA5 was closely linked to the malignancy of glioma. We also explored the relationship between MXRA5 mRNA expression level and IDH mutation status and found that MXRA5 was highly expressed in IDH wild-type glioma in both the CGGA and TCGA datasets (Figures 2(b) and 2(d)).

### 3.3. MXRA5 Protein Expression in Glioma Tissue and Cell Lines

**Figure 3:**
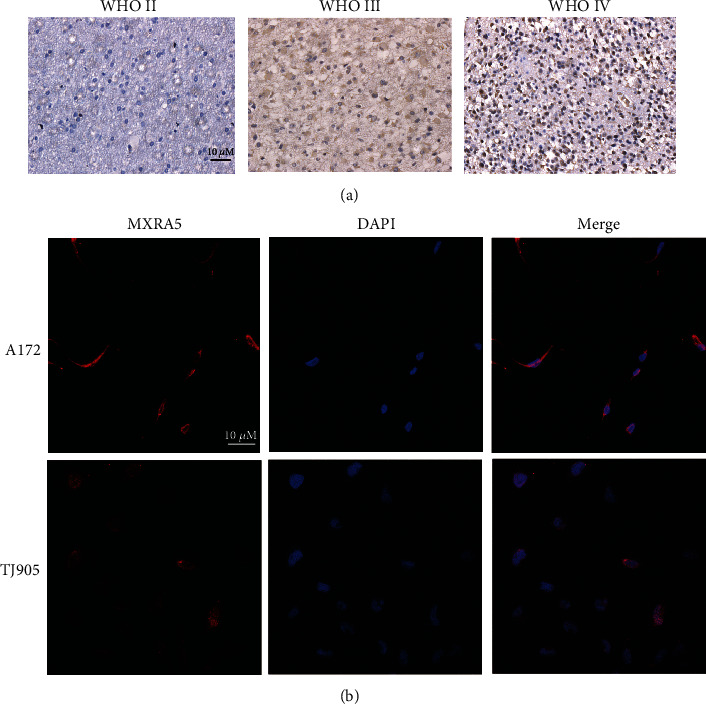
MXRA5 protein expression in glioma tissue and cell lines. (a) Immunohistochemical staining of MXRA5 in different grades of gliomas. Positive cells are stained brown. Magnification: 40x objective lens. (b) Immunofluorescence staining of MXRA5 protein (red) in the A172 and TJ905 glioma cell lines. Nuclei were stained with DAPI (blue). Magnification: 40x objective lens.

To further characterize the relationship of MXRA5 expression level and tumor specimens, we measured the MXRA5 protein level in glioma tissue samples from twenty-eight patients (1 with grade I glioma, 7 with grade II glioma, 7 with grade III glioma, and 13 with GBM) using IHC analysis. Representative IHC staining of MXRA5 in gliomas is illustrated in Figure 3(a). MXRA5 expression levels were significantly higher in GBM than in LGG (*p* < 0.05, Table 1). These results suggested that MXRA5 expression was more prevalent in aggressive glioma. To determine the subcellular localization of MXRA5, we performed IF staining with A172 and TJ905 cancer cells (Figure 3(b)). Our results showed that it could be detected in the cytoplasm.

### 3.4. MXRA5 Is a Biomarker of the Mesenchymal Subtype of GBM

**Figure 4:**
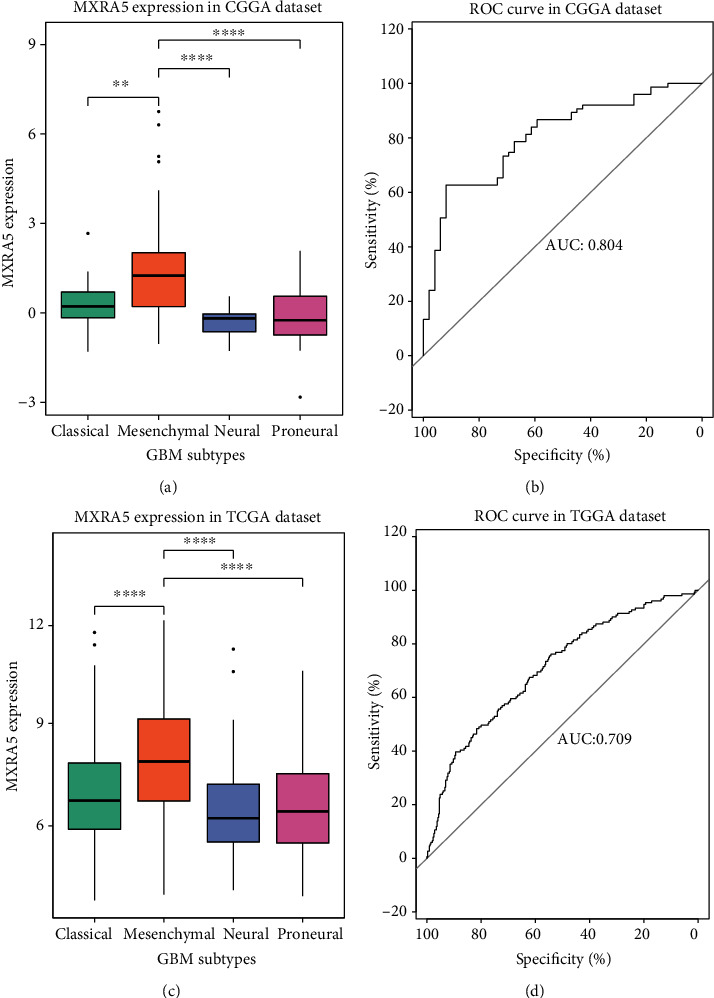
MXRA5 expression pattern in different GBM subtypes in the CGGA and TCGA datasets. (a, c) MXRA5 was significantly enriched in mesenchymal-subtype GBM. (b, d) ROC analysis showed that MXRA5 had high sensitivity and specificity for predicting mesenchymal-subtype GBM. ∗∗ and ∗∗∗∗ indicate *p* < 0.01 and *p* < 0.0001, respectively.

To investigate the relationship between MXRA5 mRNA expression and TCGA-defined molecular subtypes, we analyzed the distribution of MXRA5 mRNA expression in different TCGA molecular subtypes. The MXRA5 mRNA expression level was significantly increased in the mesenchymal subtype compared with the other three subtypes in both the CGGA and TCGA cohorts (Figures 4(a) and 4(c)). Then, we used data for MXRA5 mRNA expression in mesenchymal-subtype GBM to generate ROC curves. In the CGGA dataset, the area under the curve (AUC) was 0.804 (Figure 4(b)). In TCGA dataset, the AUC was 0.709 (Figure 4(d)). These results showed that MXRA5 was highly specifically expressed in mesenchymal-subtype GBM and may serve as a biomarker to predict mesenchymal-subtype GBM.

### 3.5. Gene Ontology (GO) and Pathway Enrichment Analysis of MXRA5-Associated Genes in GBM

**Figure 5:**
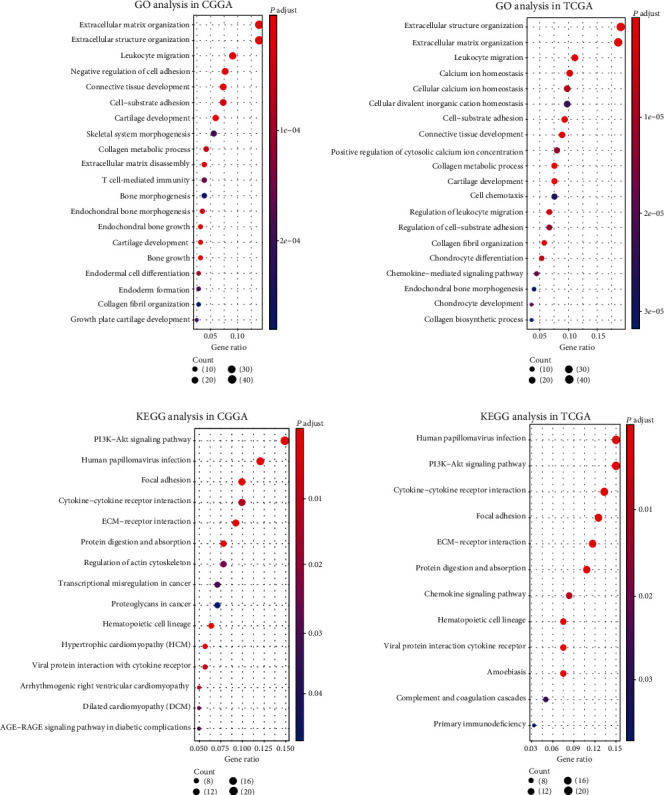
GO and KEGG pathway analyses of MXRA5-associated genes in GBM.

To explore the biological function and key pathway of MXRA5 in GBM, we performed GO analysis and Kyoto Encyclopedia of Genes and Genomes (KEGG) pathway analysis. First, we identified the genes that strongly correlated with MXRA5 by Pearson correlation analysis (Pearson  | *R* | ≥0.6 in CGGA dataset, Pearson  | *R* | ≥0.5 in TGGA dataset). Finally, 321 and 262 genes that were highly correlated with MXRA5 expression in GBM in the CGGA and TCGA datasets, respectively, were eligible for subsequent analysis. Significantly related genes were chosen for GO and KEGG analyses with R. We found that genes most relevant to MXRA5 expression were more involved in extracellular matrix organization, extracellular structure organization, and leukocyte migration in both CGGA and TCGA datasets. KEGG pathway analysis revealed that the most related genes were mainly enriched in the PI3K-AKT signaling pathway, human papillomavirus infection, and focal adhesion (Figure 5). These analyses indicate that the biological function of MXRA5 may be related to tumor invasion and the immune microenvironment of glioma.

### 3.6. The Relationship between MXRA5 and the Immune Microenvironment in GBM

**Figure 6:**
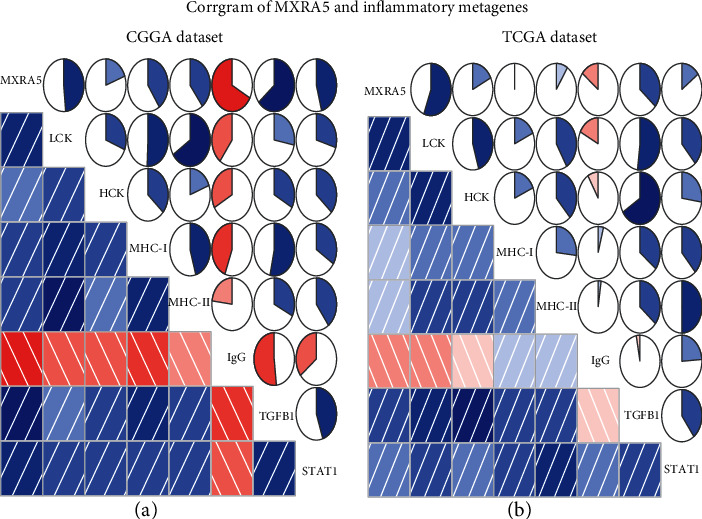
MXRA5-related inflammatory activities in GBM samples of the CGGA and TCGA cohorts. In pie charts, positive correlations are displayed in green and negative correlations are displayed in red. The color intensity and the size of the circle are proportional to the correlation coefficient.

**Figure 7:**
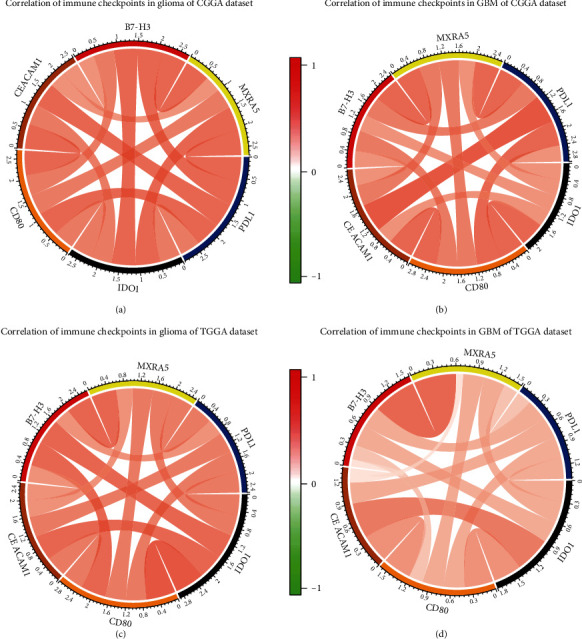
Correlation of MXRA5 and immune checkpoint molecules in glioma (a, c) and GBM (b, d).

To further clarify the role of MXRA5 in the immune response in GBM, we selected the immunosuppressive gene TGFB1 and six gene sets that represent different types of inflammation and immune response and were defined as metagenes [14]. Corrgrams were derived according to the Pearson *r* value between MXRA5 and metagenes. As shown in Figures 6(a) and 6(b), most of the metagenes were positively associated with MXRA5 expression except for IgG, which was mainly correlated with the activities of B lymphocytes. Among them, lymphocyte-specific kinase (LCK), TGFB1, and STAT1 had the highest positive correlations. Many checkpoint members that have been identified as therapeutic targets in clinical or preclinical trials and additional immune genes and immune checkpoints were included in the analysis, such as B7-H3, PD-L1, and IDO1. PD-L1 was reported to be upregulated in high-grade glioma compared with LGG. Circos plots demonstrated that MXRA5 expression was tightly associated with B7-H3 and PD-L1 (Figures 7(a)–7(d)).

### 3.7. MXRA5 Positively Correlates with Tumor-Associated Macrophages (TAMs) in GBM

**Figure 8:**
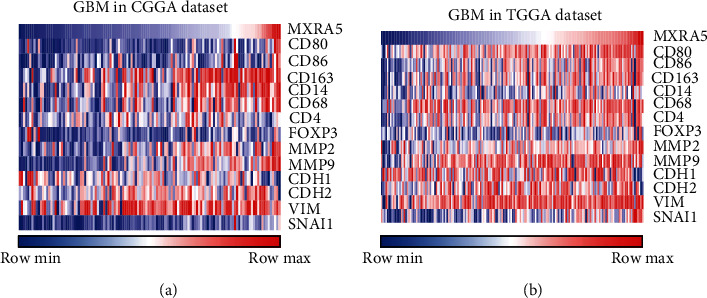
MXRA5 is positively correlated with the infiltration of tumor-associated macrophages and the expression of tumor invasion-related genes in GBM.

To further investigate the role of MXRA5 in the glioma immune microenvironment, we performed Pearson correlation analysis of key markers of TAMs and Treg cells. The analysis showed that in GBM, MXRA5 closely positively correlated with M2 tumor-associated macrophage infiltration but had little correlation with M1 and Treg infiltration (Figures 8(a) and 8(b)), which indicates that MXRA5 may play a role in tumor immunosuppression.

### 3.8. MXRA5 Is Highly Expressed in GBM and Could Predict Worse Survival in Glioma

**Figure 9:**
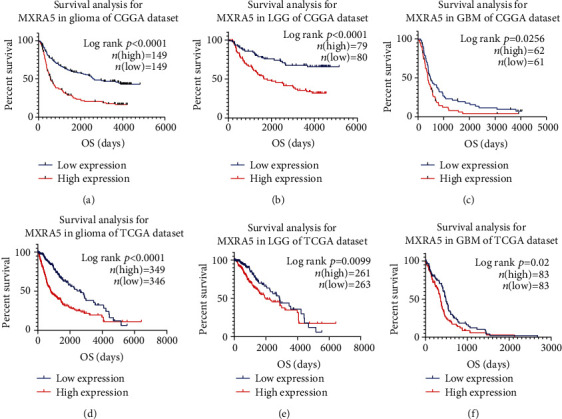
The MXRA5 survival curves of glioma and GBM in the CGGA and TCGA cohorts. Kaplan-Meier survival analysis showed that, compared to low expression, high expression of MXRA5 conferred a significantly worse prognosis in glioma and GBM patients.

To further analyze the prognostic value of MXRA5, we divided glioma patients into the MXRA5 high-expression group and low-expression group based on the MXRA5 mRNA expression level (median). As shown in Figures 9(a) and 9(d), in both CGGA and TCGA datasets, glioma patients in the high-expression group experienced significantly shorter overall survival than their low-expression counterparts. Similar Kaplan-Meier curves were significantly observed in LGG and GBM patients (Figures 9(b), 9(c), 9(e), and 9(f)).

### 3.9. MXRA5 Expression and Its Relationship with Pathological Features of Glioma

The relationship between MXRA5 mRNA expression and clinicopathological features was analyzed. Statistical analysis indicated that high mRNA expression of MXRA5 was correlated with age (*p* < 0.001), WHO grade (*p* < 0.001), and IDH mutation status (*p* < 0.001). No significant correlation was found between MXRA5 mRNA expression and sex (*p* = 0.125), MGMT promoter methylation status (*p* = 0.118), radiotherapy (*p* = 0.156), or chemotherapy status (*p* = 0.965) (Table 2). Furthermore, univariate and multivariate Cox regression hazard analyses showed that the MXRA5 mRNA expression level was an independent prognostic factor for glioma (*p* = 0.018) (Table 3).

## 4. Discussion

The prognosis of glioma is poor, and numerous traditional antitumor drugs fail to prolong the survival time of patients with glioma, especially GBM [15]. The rise of immune and targeted therapy has brought new hope to patients. At present, a series of biomarkers have been identified in local tumor tissues, but circulating markers that could be detected in body fluids are still lacking [16]. For a long time, we have been committed to finding suitable therapeutic targets through bioinformatics analysis.

MXRA5, a secreted adhesion proteoglycan with VEGF receptor activity, is elevated in the cartilage of patients with osteoarthritis and can be detected in synovial fluid [17, 18]. Moreover, it is upregulated by TGF*β*-1 in chronic renal tubular disease and involved in the progression of chronic renal disease to end-stage renal disease. Studies have shown that MXRA5 also plays an important role in the development and progression of cancers [19–22]. However, the prognostic value and role of MXRA5 in glioma remain unclear.

In the present study, we found that MXRA5 mRNA and protein expression was closely related to tumor grade, with the highest expression in GBM among other glioma subtypes, and the expression in mesenchymal-subtype GBM was higher than that in other subtypes. Mesenchymal-subtype GBMs are more invasive and have a worse prognosis than other subtypes [23]. MXRA5 is highly expressed in mesenchymal-subtype GBM and can be used as a predictor. This suggests that MXRA5 is a marker of poor prognosis. Analysis of clinical survival data also confirmed that the survival time of glioma patients with high expression of MXRA5 was significantly shorter than that of patients with low expression. This prognostic value was also observed in patients with GBM.

IDH mutation status is of great significance to the molecular classification of glioma and significantly affects the prognosis of patients. The prognoses of IDH wild-type glioma and GBM are worse than those of IDH mutant glioma and GBM [24, 25]. However, the molecular mechanisms are not well understood. Our results suggest that MXRA5 is more highly expressed in IDH wild-type glioma, which indirectly suggests that MXRA5 is more highly expressed in glioma with poor prognosis. In addition, IDH wild-type glioma may also regulate the occurrence and progression of the tumor through the MXRA5 pathway, leading to poor prognosis.

Additionally, Cox univariate and multivariate analyses of MXRA5 expression and clinicopathological features revealed that MXRA5 expression has a correlation with age and WHO pathological stage, suggesting that MXRA5 could be an independent prognostic factor for glioma patients. These results indicate that MXRA5 is involved in the malignant biological process of glioma. However, the specific mechanism by which MXRA5 mediates glioma development remains unclear.

Minafra et al. confirmed that MXRA5 was involved in epithelial-mesenchymal transition (EMT) and matrix remodeling in breast cancer [26]. Ding et al. found that MXRA5 expression is decreased in preeclampsia and affects trophoblast cell invasion through the MAPK pathway [27]. Through CGGA and TCGA transcriptome analysis, we found that the expression of MXRA5 was highly positively correlated with the expression of immune checkpoints, the immunosuppressive factor TGFB1, and tumor invasion markers. TAMs are divided into M1 and M2 macrophages, which are important immune cells in the glioma microenvironment. M1 macrophages express high levels of cluster of differentiation (CD) 80 and CD86, which are reportedly antitumoral and are associated with better prognosis. M2 macrophages are defined by high cell surface levels of CD163, CD204, and CD14 and mediate the immunosuppressive response [28]. The majority of glioma-associated microglia/macrophages have been identified as M2 macrophages with immunosuppressive and tumor supportive action. We performed Pearson correlation analysis of key markers of TAMs and Treg cells. The analysis showed that in GBM, MXRA5 expression closely correlated with M2 tumor-associated macrophage infiltration but had little correlation with M1 and Treg infiltration. Therefore, we hypothesized that MXRA5 is likely to promote the malignant progression of GBM by participating in the immunosuppression of the tumor microenvironment. However, the exact molecular mechanism remains to be clarified through further experimental research.

The study of biomarkers has always been a hot spot in the field of glioma. However, until now, clinically significant circulating markers have been scarce. Sreekanthreddy et al. [29] found that the expression levels of OPN and TIMP1 in serum of GBM patients were higher than those in LGG patients and the survival time of GBM patients with higher OPN expression level was shorter. It has been reported that there are many kinds of molecules in the cerebrospinal fluid of glioma patients, such as IL-6 and miR-21, that have significantly higher levels than those in normal people [30, 31]. However, due to the lack of sensitivity and specificity, there is still a lack of ideal circulating markers. As a secreted glycoprotein, MXRA5 is mainly expressed in the extracellular stroma and cytoplasm and can be detected in body fluids such as articular fluid and urine [18, 32]. Therefore, MXRA5 could be a potential circulating marker.

However, our study had several limitations. The analysis in our study was mainly based on the transcriptional level of MXRA5, and the protein level of MXRA5 was only preliminarily detected by immunohistochemistry. The direct relationship between MXRA5 protein levels and patient outcomes needs to be further verified. In addition, the role of MXRA5 in glioma was only preliminarily speculated through bioinformatics analysis. These hypotheses need to be tested in real molecular biology experiments.

In conclusion, our study has shown that MXRA5 is aberrantly expressed in mesenchymal-subtype GBM and that high MXRA5 expression indicates poor overall survival. These results reveal the potential value of MXRA5 as a novel biomarker and immunotherapy target. We will continue to explore the molecular mechanism by which MXRA5 influences glioma biology.

## Figures and Tables

**Table 1:** MXRA5 immunohistochemical expression in 28 glioma tissue samples.

Samples	*N*	Expression of MXRA5		*p* value
		Positive	Negative	
LGG	15	2 (13.3%)	13 (86.6%)	0.042
GBM	13	7 (53.8%)	6 (46.2%)	

**Table 2:** Correlation between the MXRA5 expression and the clinicopathologic characteristics of glioma patients in the CGGA dataset.

Variable	MXRA5 expression		*p* value
	Low (*n*)	High (*n*)	
Age			<0.001
≥45	42	80	
<45	105	69	
Sex			0.125
Male	82	95	
Female	67	54	
WHO grade			<0.001
GBM	40	83	
LGG	109	66	
IDH			<0.001
Mutation	93	42	
Wild type	56	107	
MGMT promoter			0.118
Unmethylated	89	97	
Methylated	57	42	
Radiotherapy			0.156
Untreated	19	27	
Treated	125	112	
Chemotherapy			0.965
Untreated	74	70	
Treated	68	65	

GBM: glioblastoma multiforme; LGG: lower-grade glioma; IDH: isocitrate dehydrogenase; MGMT: methylguanine methyltransferase.

**Table 3:** Univariate and multivariate analyses of clinical prognostic parameters in the CGGA dataset. MXRA5 expression was an independent prognostic factor in the CGGA dataset.

Variable	Univariate analysis		Multivariate analysis	
	HR (95% CI)	*p* value	HR (95% CI)	*p* value
MXRA5 expression	2.310 (1.717-3.107)	<0.001	1.491 (1.071-2.077)	0.018
	

Age at diagnosis	2.079 (1.555-2.779)	<0.001	1.376 (1.011-1.873)	0.042
	

WHO grade	4.552 (3.356-6.176)	<0.001	3.402 (2.382-4.857)	<0.001
	

IDH mutation	2.524 (1.859-3.426)	<0.001	1.212 (0.832-1.765)	0.317
	

## Data Availability

The data used to support the findings of this study are included within the article.
